# Myb-Binding Protein 1A (MYBBP1A) Is Essential for Early Embryonic Development, Controls Cell Cycle and Mitosis, and Acts as a Tumor Suppressor

**DOI:** 10.1371/journal.pone.0039723

**Published:** 2012-10-08

**Authors:** Silvia Mori, Rosa Bernardi, Audrey Laurent, Massimo Resnati, Ambra Crippa, Arianna Gabrieli, Rebecca Keough, Thomas J. Gonda, Francesco Blasi

**Affiliations:** 1 Università Vita Salute San Raffaele, Milan, Italy; 2 San Raffaele Scientific Institute, Milan, Italy; 3 Fondazione Istituto FIRC di Oncologia Molecolare (IFOM), Milano, Italy; 4 Flinders University, Bedford Park, Adelaide, South Australia, Australia; 5 University of Queensland Diamantina Institute, Brisbane, Queensland, Australia; 6 Division of Human Immunology and Hanson Institute, Institute of Medical and Veterinary Science, Adelaide, South Australia, Australia; Lerner Research Institute, United States of America

## Abstract

MYBBP1A is a predominantly nucleolar transcriptional regulator involved in rDNA synthesis and p53 activation via acetylation. However little further information is available as to its function. Here we report that MYBBP1A is developmentally essential in the mouse prior to blastocyst formation. In cell culture, down-regulation of MYBBP1A decreases the growth rate of wild type mouse embryonic stem cells, mouse embryo fibroblasts (MEFs) and of human HeLa cells, where it also promotes apoptosis. HeLa cells either arrest at G2/M or undergo delayed and anomalous mitosis. At mitosis, MYBBP1A is localized to a parachromosomal region and gene-expression profiling shows that its down-regulation affects genes controlling chromosomal segregation and cell cycle. However, MYBBP1A down-regulation increases the growth rate of the immortalized NIH3T3 cells. Such Mybbp1a down-regulated NIH3T3 cells are more susceptible to Ras-induced transformation and cause more potent Ras-driven tumors. We conclude that MYBBP1A is an essential gene with novel roles at the pre-mitotic level and potential tumor suppressor activity.

## Introduction

The 160-kDa Myb-binding protein 1a (MYBBP1A, also referred to as p160) is a nucleolar protein whose function is mostly unknown. Although predominantly localized in the nucleolus, MYBBP1A is found also in the nucleoplasm [1]; indeed it contains nuclear and nucleolar localization sequences as well as export signals in the C-terminus [2],[3]. Unlike full length MYBBP1A/p160, its shorter N-terminal 140 KDa and 67 kDa forms are mainly localized in the nucleoplasm due to processing of the nucleolar localization signal at the C-terminus. MYBBP1A processing is regulated by stress signals affecting ribosomal biosynthesis, which induce its translocation from the nucleolus to the nucleoplasm [3], [4].

The ubiquitously expressed MYBBP1A was originally identified as an interactor of the c-Myb oncoprotein with homology to the fifth essential DNA polymerase of *S. cerevisiae* [1], [5]. MYBBP1A binds to and inhibits several transcription factors, such as the PPARγ co-activator 1α (PGC-1α) [6]. In addition, it binds the RelA/p65 subunit of NF-κB and suppresses its *trans*-activating capacity by competing with p300 [7]. MYBBP1A also binds the developmentally essential Prep1 homeodomain transcription factor and inhibits its activity by competing with its DNA-binding partner Pbx1 [4]. MYBBP1A is also a component of the Ret–CoR co-repressor complex [8]. In contrast to these inhibitory activities, MYBBP1A binds to and stimulates the aryl hydrocarbon receptor (AhR) [9]. Finally, MYBBP1A is stabilized by Prep1 at least in the skeletal muscle, and its decrease in the *Prep1*-hypomorphic mice is responsible for the hyper-activation of PGC-1α and increased insulin sensitivity [10].

MYBBP1A is also required for the acetylation and subsequent stabilization of p53 since it prevents Mdm2-dependent degradation of p53 [11]. Inhibition of p53 acetylation blocks Cdkn1a (p21) transcription and induces cell cycle arrest and apoptosis [12], [13]. Finally, MYBBP1A has also been linked to mitosis, since it is a substrate of Aurora-B kinase [14].

These multiple and diverse functions of MYBBP1A suggest that this protein plays an important role in essential biological functions such as cell division, cell proliferation and apoptosis. However nothing is known of its importance relative to other well-known regulators of these pathways, nor about its physiological function during development. Indeed, at least some functional interactors of MYBBP1A, such as p53, are not essential during development. It is clear, therefore that *in vivo* data are essential to fully evaluate the function of MYBBP1A.

In this paper we report that *MYBBP1A* is essential for early mouse development prior to blastocyst formation. We also report that *MYBBP1A* down-regulation induces apoptosis and mitotic anomalies in mouse embryonic stem cells, embryonic fibroblasts and human HeLa cells, where it alters the expression of key cell cycle regulators. Moreover, Mybbp1a may act as a tumor suppressor, as its down-regulation facilitates the transformation of NIH3T3 cells by the *HRas* oncogene.

## Materials and Methods

### Generation of Mybbp1a-targeted mice

The mice were housed in the University of Adelaide Medical School Animal House and all mouse work was covered by ethics approval from both the Institute of Medical and Veterinary Science and the University of Adelaide animal ethics committees. This project was covered by University of Adelaide Animal Ethics Committee approvals M6098 and M5201 and IMVS Animal Ethics Committee project no. 69/98.

The *Mybbp1a* targeting vector was constructed from sequences subcloned from an E14TG2a ES cell genomic library. A 3.8 kb 5′ genomic fragment containing the promoter region approximately 450 bp upstream from the initiation ATG was generated by PCR amplification using oligos (KO1: 5′-ATAAGAATGCGGCCGCTCTGTCCATTGTTGTTCTGC-3′; KO2:5′-GCTCTAGAATGTAGGGAGCAGAGGGAGCC-3′) incorporating NotI and XbaI sites at the 5′ and 3′ ends respectively. A 6.0 kb XbaI/HindIII 3′ genomic fragment encompassing part of exon 14, exons 15–26 and 3′ flanking sequences was cloned downstream of the 5′ fragment. The 1.8 kb pgkNEO expression cassette [15] was inserted into the XbaI site between the two *Mybbp1a* genomic fragments in the opposite transcriptional orientation to the *Mybbp1a* gene (**Suppl. Figure S1**). The *Mybbp1a* targeting vector was electroporated into the W9.5 ES cell line and selected with G418 as described [16]. Homologous recombinants were identified by Southern analysis of XbaI-digested DNA isolated from individual clones. Southern blots were hybridized with the 5′ genomic DNA probe (**Suppl. Figure S1**), a 950 bp fragment located 60 bp upstream of the 5′ end of the targeted region. This probe was generated by PCR amplification using the oligonucleotides: 5′-CTTGGGTCTTCTTGGGGTTCC-3′; 5′-GCCAGCATGGCAGGCTTGG-3′. The targeted deletion was confirmed by further analysis of Southern analysis of selected clones with the 3′ genomic DNA probe (**Suppl. Figure S1** and below). Targeted clones were injected into C57BL/6 blastocysts. Germline transmission was seen for a single clone and chimeric offspring were bred to C57BL/6 mice to derive heterozygous *MYBBP1A* ^+/−^ mice.

### Analysis of *Mybbp1a* ^−/+^×*Mybbp1a* ^−/+^ crosses

Chimeric mice and their heterozygous progeny were backcrossed for at least six generations onto a C57BL/6 background. Mouse genotyping was performed on tail DNA of weaned mice and E11.5 embryos prepared using DNeasy kits (Qiagen), Southern blot analysis of XbaI-digested DNA and probing with the 5′ probe as described above and shown in **Suppl. Figure S1** B, C. Mouse embryos were genotyped by PCR using oligonucleotides KO3 and KO4 to detect the wild type allele (488 bp product) and KO3 and KO5 to detect the targeted allele (570 bp product) as shown in **Suppl. Figure S1** D. (KO3: 5′-AAGGCTCCCTCTGCTCCCTAC-3′; KO4: 5′-GGCTCTTCATCTCCGCCATGC-3′; KO5: 5′-GCGCATCGCCTTCTATCGCC-3′). DNA from E9.5, E6.5 embryos and from blastocyst outgrowths was prepared by boiling at 100°C for 8–10 min in water∶PBS (1∶1). Blastocysts were cultured in DMEM containing 10% FCS for 4–6 days and outgrowth material was collected for PCR analysis as above.

### Cell culture and treatment

Wild-type ES cells were derived from E2.5–3.5 C57Bl/6 embryos using standard procedures [17]. Cells were cultured in LIF-containing medium (DMEM+GlutaMAX™-I, Gibco) complemented with 15% Fetal Calf Serum, 0.1 mM nonessential aminoacids, 1 mM sodium pyruvate and 0.1 mM β-Mercaptoethanol, on 0.2% gelatin-coated dishes. After *Mybbp1a* -shRNA or empty vector lentiviral infection and puromycin selection (1 µg/mL), 1.25×10^5^ cells per well were plated in 6-well plates and cultured. Cells were harvested and counted in triplicate on days 1, 2 and 3.

MEFs were derived from wt C57BL/6 embryos using standard procedure.

NIH3T3, MEFs, HeLa and Phoenix cells were grown at 37°C, 5% CO_2_ in Dulbecco's modified Eagle's medium (DMEM, Gibco) supplemented with 10% heat-inactivated fetal bovine serum (FBS, Euroclone), 0.2 mg/ml streptomycin (Gibco), 20 U/ml penicillin (Gibco), 2 mM glutamine (Gibco) and 1 mM sodium pyruvate (Gibco). HEK 293T were grown in Iscove's Modified Dulbecco's medium (IMDM, Cambrex) supplemented with 10% FBS, 0.2 mg/ml streptomycin, 20 U/ml penicillin and 2 mM glutamine.

To block cells at the G1/S transition, a double-Thymidine block was used. Cells (NIH 3T3 or HeLa) were treated with 2 mM Thymidine (Sigma) for 18 hours (first block), followed by a release of 9 hours and a second pulse with 2 mM Thymidine for 17 h (second block).

To transiently silence *MYBBP1A* in HeLa cells, 2×10^5^ cells/well were plated in triplicate in 6-well plates for 24 hours, transfected using lipofectamine (Invitrogen) with 40 nM siRNA or control oligonucleotides (High-GC, Medium-GC, Invitrogen), following the manufacturer protocol. The sequences of the siRNAs are the following:

siRNA1 (CCAGGCTGGTGAATGTGCTGAAGAT, 52% GC);

siRNA2 (CCCTGCAGCTAATTCTGGATGTGCT, 52% GC);

siRNA3 (GAGGTCCTCAAAGCCGACTTGAATA, 48% GC).

For retroviral infection and selection of stable clones, Phoenix ecotropic packaging cells [18] were transfected with 10 µg of retroviral plasmid using the calcium phosphate protocol [19] and incubated overnight. Two days post- transfection NIH 3T3 cells were infected with the filtered viral supernatant and supplemented with 8 µg/ml of Polybrene (Sigma) for 4 h at 37°C. Antibiotics were added 24 h post infection for the selection: Geneticin for pRufNeo-p160-Flag (1 mg/ml, Gibco) [20] and Hygromycin for pBabe-Myc and –RasVal12 (200 µg/ml, Invitrogen).

For lentiviral infection and selection of stable clones, HEK 293T cells were transfected with 3.5 µg ENV plasmid (VSV-G), 5 µg packaging plasmid (pMDLg/p RRE), 2.5 µg of pRSV-REV and 15 µg of the target plasmid (pLKO.1-puro, containing one of the five different shRNA target sequences, Sigma) following a calcium phosphate transfection protocol. The day after transfection the medium was replaced by fresh medium supplemented with 1 mM Sodium Butyrate (Sigma). After 30 h, the viral supernatant was collected and used to infect NIH 3T3 cells. The day after the infection 2 µg/ml Puromycin was added to the medium for selection. The shRNA target sequences are the following:

shRNA1 (CCGGCCTGCCCTAGAGACTCCTATTCTCGAGAATAGGAGTCTCTAGGGCA); shRNA2 (CCGGCCTGATGAAGTCCGTGCAATTCTCGAGAATTGCACGGACTTCATCA); shRNA3 (CCGGCCGGAGTGTATTTGGTCATATCTCGAGATATGACCAAATACACTC); shRNA4 (CCGGCCCAATGATTCGGAGATGAAACTCGAGTTTCATCTCCGAATCATTG); shRNA5 (CCGGCCAAGCGTAACAGCTCACTTACTCGAGTAAGTGAGCTGTTACGCTT).

As controls, commercial High-GC and Medium-GC content shRNAs (Invitrogen) were used as suggested by the manufacturer.

### Proliferation assay

Measurement of HeLa cell proliferation was performed by plating 2×10^5^ cells/well in a 6-well plate and transfecting them with lipofectamine the day after. At 24, 48 and 72 h after transfection the cells were detached and counted with a cell counter (Coulter Z1, Coulter Diagnostics).

Crystal Violet assay was performed plating 3,000 cells/well in a 12-well plate and fixing the cells at the following time points: T0 (8 h after plating) and 2, 4, 6, 8, 10 days after. Cells were washed in PBS, fixed with 11% glutaraldehyde (Sigma) for 15 min. at RT, washed again in PBS and allowed to dry at RT. Cells were stained with Crystal Violet solution (0,1% crystal violet -Sigma- in 20% Methanol, 80% dH_2_O) for 20 min. at RT. Cells were washed with dH_2_O and dried at RT. Stained and dried cells were solubilized with a solution of 10% Acetic Acid for 10 min. at RT and analyzed with a Microplate Reader 680 (Bio-Rad) at 570 nm.

Long term proliferation experiments were performed by plating 300000 cells in 6-well plates. After 3 days, cells were counted with a cell counter (Coulter Z1, Coulter Diagnostics) and plated again at 300000 cells per well and counted again the third day. The procedure was repeated 5 times.

### Immunoblot analysis

Cells were washed with PBS 1× and scraped with RIPA buffer (100 µl for 10 cm dish; buffer composition: 5 mM Tris-HCl pH 8, 150 mM NaCl, 0.1%, SDS 1% NP-40, 0.5%, Na-deoxycholate, 1× Complete Protease Inhibitor, EDTA free, Roche). Cells were incubated on ice for 30 min and after centrifugation at 14,000 rpm for 15 min at 4°C, protein concentration was detected with the Bradford reagent (Bio-Rad Protein Assay, Bio-Rad) and measurement at 595 nm with Ultraspectrophotometer 2100pro. Protein extracts were resuspended in Laemmli buffer [30], heated for 5 minutes at 95°C, loaded on a 10% gradient sodium dodecyl sulfate-polyacrylamide gel electrophoresis (SDS-PAGE) and immunoblotted with the indicated antibodies.

ES cell immunoblotting analysis was performed with anti-Oct-3/4 (C-10) (1∶500, Santa Cruz), anti-Cleaved Caspase-3 (Asp175) (1∶1000, Cell Signalling), anti p160, and anti-Vinculin (1∶5000, Sigma).

### Immunocytochemical analysis and confocal microscopy

Cells were grown on sterile 13 mm glass coverslips (VWR international) for at least 24 h. Cells were washed with PBS and fixed (3% paraformaldehyde; 2% sucrose in 1× PBS) for 10 min at RT. After washing with PBS, cells were permeabilized with 0.2% PBS-Triton X-100 for 5 min at RT, washed with PBS and blocked with 1× PBS-1% BSA for 30 min at RT. Coverslips were incubated with the indicated antibodies in blocking solution for 30 min at 37°C. Cells were washed with PBS and incubated with the secondary antibodies (Alexa-488 and Alexa-546 conjugated anti-mouse or anti-rabbit immunoglobulin) and incubated for 30 min at RT protected from light. Cells were washed again with PBS and incubated with 4′6-diamidino-2-phenylindole dihydrochloride (DAPI) nuclear staining (Fluka) for 3 min at RT or Hoechst 33258 (same conditions, Sigma). Cells were then washed with PBS and coverslips mounted with Fluorescent Mounting Medium (Dako Cytomation). Images were obtained with a Leica DMIRE2 (Confocal System Leica TCS SP2) with a 63× objective. Incubations with matched mouse isotype IgGs, irrelevant rabbit IgGs or secondary antibodies were always negative.

### ImmunoFISH

After the washing step following the incubation with the secondary antibodies, cells were fixed again in 4% paraformaldehyde, 0.1% Triton X-100 at 4°C for 30 minutes. Cells were then incubated for 30 min at RT with 100 mM Glycine (VWR) and washed once with PBS. FISH was performed with Telomere PNA FISH kit/Cy3 (DAKO) following the manufacturer instructions; PNA probe was labeled with Cy3.

### Time-Lapse microscopy

Cells were plated in a 6-well plate (2×10^5^ cells/well in triplicate), transfected as described above with siRNA or High-GC control and blocked at the G1/S transition using a double thymidine block. Starting 8 h after the release from the block and up to 24 hours later, the cells were analyzed with Time Lapse Microscope (OKO VISION). Images were taken in 4 different fields for each sample every 5 min with a 5× objective and analyzed with Image J software.

### Flow Cytometry analysis

For 5′ethynyl-2′-deoxyuridine (EdU)/7-amino-actinomycin D (7-AAD) double staining, cells were grown and pulsed before the assay with 10 µM EdU for 1 hour, harvested and processed with Click-iT EdU Flow Cytometry Assay kit (Invitrogen) following the manufacturer protocol. Anti-EdU-Fluorescein-isothycyanate (FITC) was used to stain EdU positive cells and 7-AAD was used to stain DNA. Cells were analyzed with FACS CANTO or FACS Calibur (BD Immunocytometry). Results were displayed as bivariate distribution of EdU content versus DNA content. The percent of cells in the S-phase was calculated by gating EdU positive cells using FCS Express V3 (De Novo Software) or CellQuest program (BD).

For Annexin V staining, cells were harvested, washed with PBS 1× and the dead and apoptotic cells were detected by Annexin V Phycoerythrin (PE)/7-AAD staining using PE Annexin V Apoptosis Detection kit I (BD Pharmingen), following the manufacturer's instructions. Cells were analyzed with FACS CANTO or Calibur (BD) and results were displayed as bivariate distribution of Annexin V positive cells versus 7-AAD positive cells. The percentage of early apoptotic cells was calculated by gating Annexin V positive cells using FCS Express V3 or CellQuest programs.

### Gene expression profiling

#### Real-Time PCR

Total RNA was extracted (RNAeasy minikit, QIAGEN) and quantified by spectrophotometry (Nanodrop, Thermo Scientific). Total RNA (5 µg) was reverse-transcribed using a SuperScript First-Strand kit with random primers (Invitrogen) according to the manufacturer's instructions. For quantitative Real-Time PCR, 20 ng of the reverse-transcribed RNA was amplified with a Light-Cycler 480 (Roche) using a Universal Probe Library Assay (Roche) and following the manufacturer instructions. The PCR conditions were as follow: pre-incubation at 95°C for 5 minutes, 50 cycles of amplification (denaturation at 95°C for 10 s, annealing at 58°C for 15 s and extension at 72°C for 1 s), cooling at 40°C for 10 s. The amount of the different mRNAs was normalized to GAPDH mRNA. The sequences of the primers used with the relative probes are the following: *MYBBP1A* (probe 10, L: AGCACCTTCTGCTCCTCGT, R: ATGCAGGTCTGGATGTCACC); CDKN1A (probe 32, L: TCACTGTCTTGTACCCTTGTGC, R: GGCGTTTGGAGTGGTAGAAA); NFKB2 (probe 10, L: CACATGGGTGGAGGCTCT, R: ACTGGTAGGGGCTGTAGGC); GADD45B (probe 10, L: CATTGTCTCCTGGTCACGAA, R: TAGGGGACCCACTGGTTGT); JUN (probe 19, L: CCAAAGGATAGTGCGATGTTT, R: CTGTCCCTCTCCACTGCAAC); TOP2B (probe 66, L: TCCAAGAGATTCTTTGCTTAGGA, R: CATCCTCTTCTTCTGAGAAATCAAA; GAPDH (probe 60, L: AGCCACATCGCTCAGACAC, R: GCCCAATACGACCAAATCC). The following genes were analysed with Applied Biosystems probe sets: CKS1b (probe Hs01029137_g1); SFN (probes Hs00602835_s1 Hs00968567_s1); TOP2A (probe Hs00172214_m1; WEE1 (probe Hs00268721_m1); BRCA1 (probe Hs00173233_m1); CDK7 (probe Hs00361486_m1).

RNAs from three technical replicates of HeLa cells transfected with High-GC control or siRNA1, were analyzed with Affimetrix Human Gene 1.0 ST chip (Affimetrix). Biotin-labelled cDNA targets were synthesized starting from 100 ng of total RNA. Double stranded cDNA synthesis and related cRNA was obtained with GeneChip WT cDNA Synthesis and Amplification Kit (Ambion). With the same kit the cDNA sense strand was synthesized, then fragmented and labelled with GeneChip WT Teminal Labeling Kit (Ambion). DNA fragmentation was performed with a combination of uracil DNA glycosylase (UDG) and apurinic/apyrimidinic endonuclease 1 (APE 1), while labeling was completed using terminal deoxynucleotidyl transferase (TdT) in the presence of GeneChip® DNA Labeling Reagent. All steps of the labelling protocol were performed as suggested by Affymetrix (http://www.affymetrix.com/support/technical/manual/expression_manual.affx). The size and the accuracy of quantification of targets were checked by agarose gel electrophoresis of 2 µl aliquots of each sample after fragmentation. External controls (spikes) were used; each eukaryotic GeneChip® probe array contains probe sets for several B. subtilis genes that are absent in the samples analyzed (lys, phe, thr, and dap). This Poly-A RNA Control Kit contains *in vitro* synthesized, polyadenylated transcripts for these B. subtilis genes that are pre-mixed at staggered concentrations to assess the overall success of the assay. Samples were then hybridized as indicated by Affymetrix. A single GeneChip Human Gene 1.0 ST was then hybridized with each biotin-labelled sense target. Hybridizations were performed for 16 h at 45°C in a rotisserie oven. GeneChip cartridges were washed and stained with GeneChip Hybridization, Wash and Stain Kit in the Affymetrix fluidics station following the FS450_0007 standard protocol. Images were scanned using an Affymetrix GeneChip Scanner3000 7G with default parameters. The resulting images were analysed using GeneChip Operating Software v1.2 (GCOS1.2). Raw data were first normalized using RMA method [21]–[23]. In order to evaluate the variability between samples, Principal Component Analysis (PCA) was performed. After this analysis an ANOVA test and a False Discovery Rate (FDR) [24], [25] to exclude false positives) was applied and the resulting gene-list was refined with a p value≤0.05 and a fold change ≥1.75. Gene ontology analyses of the differentially expressed genes were performed with the DAVID 6.7 functional annotation tool (Database for Annotation, Visualization and Integration Discovery – NIH, http://david.abcc.ncifcrf.gov/home.jsp) according to [26].

### Colony forming assay

Cells were resuspended in DMEM with 0.3% agar at a concentration of 5,000 cells/well and plated over a 0.6% agar layer in a 6-well plate. The experiment was performed in triplicate and plates were incubated at 37°C in a humidified incubator for two weeks. Colony detection was performed by staining cells with 3-(4,5-dimethylthiazol-2-ye)-2,5-diphenyltetrazolium bromide (MTT, 1∶10 in PBS 1×) for at least 4 h. After staining, the plate was scanned and the image obtained was analyzed with Image J Software.

### Tumorigenesis in nude mice

NIH 3T3 cells were infected with either an lentivirus empty vector (NT) or a vector expressing specific *Mybbp1a* shRNA (Sh3, see above) and selected with puromycin (4 µg/mL). After selection, stable puromycin-resistant pools were subsequently infected with pBABE-RasV12 or empty pBABE vector (as indicated), selected with 50 mg/mL hygromycin and 4×10^6^ cells for each condition were injected in nude mice (6 mice per group). After the injection, mice were monitored daily for 18 days and tumor volume measured every 2 days.

### Antibodies

The following antibodies were used: mono- and polyclonal antibodies against Flag (Sigma-Aldrich, St. Louis, MO, USA); polyclonal antibody against p160C (Zymed laboratories Inc., South San Francisco, CA, USA); mouse monoclonal antibody against Prep1 (Santa Cruz Biotechnology, Santa Cruz, CA, USA); mouse monoclonal antibody against Tubulin (Sigma); rabbit polyclonal antibody against Phospho Histone H3 (Millipore Upstate Biotechnology, NY, USA); human monoclonal antibody against CREST (homemade); mouse monoclonal antibody against Nucleolin (Santa Cruz Biotecnology); mouse monoclonal antibody against Nucleophosmin (Invitrogen, Camarillo CA, USA); rabbit polyclonal antibody against active Caspase3 and mouse monoclonal antibody against Caspase 9 (MBL, Woburn, MA, USA).

## Results

### Deletion of *Mybbp1a* in the mouse leads to embryonic death prior to blastocyst formation

The experimental approach used to knock out the mouse *Mybbp1a* gene is shown in **Supplementary Figure S1** (see also Materials and Methods). The *Mybbp1a* targeting vector comprised 3.5 kb of 5′ genomic flanking sequence and 6.0 kb of genomic sequence encompassing part of exon 14, exons 15–26 and 3′ flanking sequences. Recombination resulted in the deletion of the 5′ half of the coding region of the gene (5.5 kb) including the ATG, exons 1 to 13 and a half of exon 14 (**Suppl. Figure S1A**). Healthy and fertile heterozygous *Mybbp1a* ^+/−^ mice were obtained. However heterozygous crosses yielded no homozygous knockout mice, indicating that *Mybbp1a* is essential for mouse development (**Table 1**). Analysis of embryos at various times of development yielded no homozygous *Mybbp1a* ^−/−^ embryos at E11.5 (p<0.05), E9.5 (p<0.05) nor at E6.5 (p<0.05). Blastocysts were then isolated from a *Mybbp1a* ^+/−^ heterozygous cross, grown in culture for several days and genotyped. No *Mybbp1a* −/− blastocysts were detected (**Table 1**). These results strongly indicate that *Mybbp1a* is essential during the very early stages of embryonic development before blastocyst formation.

**Table 1:** Timed pregnancy analysis of heterozygous *MYBBP1A* ^+/−^ crosses.

Time of analysis	N	N+/+	N+/−	N−/−	NNP	p-value
**Birth**	61	11	50	0	0	<0.05
**E11.5**	18	5	13	0	0	<0.05
**E9.5**	22	7	15	0	0	<0.05
**E6.5**	28	10	15	0	3	<0.05
**Blastocyst**	30	8	20	0	2	<0.05

N = Total number.

NP = No PCR product.

### MYBBP1A is required for proper cell growth

**Figure 1:**
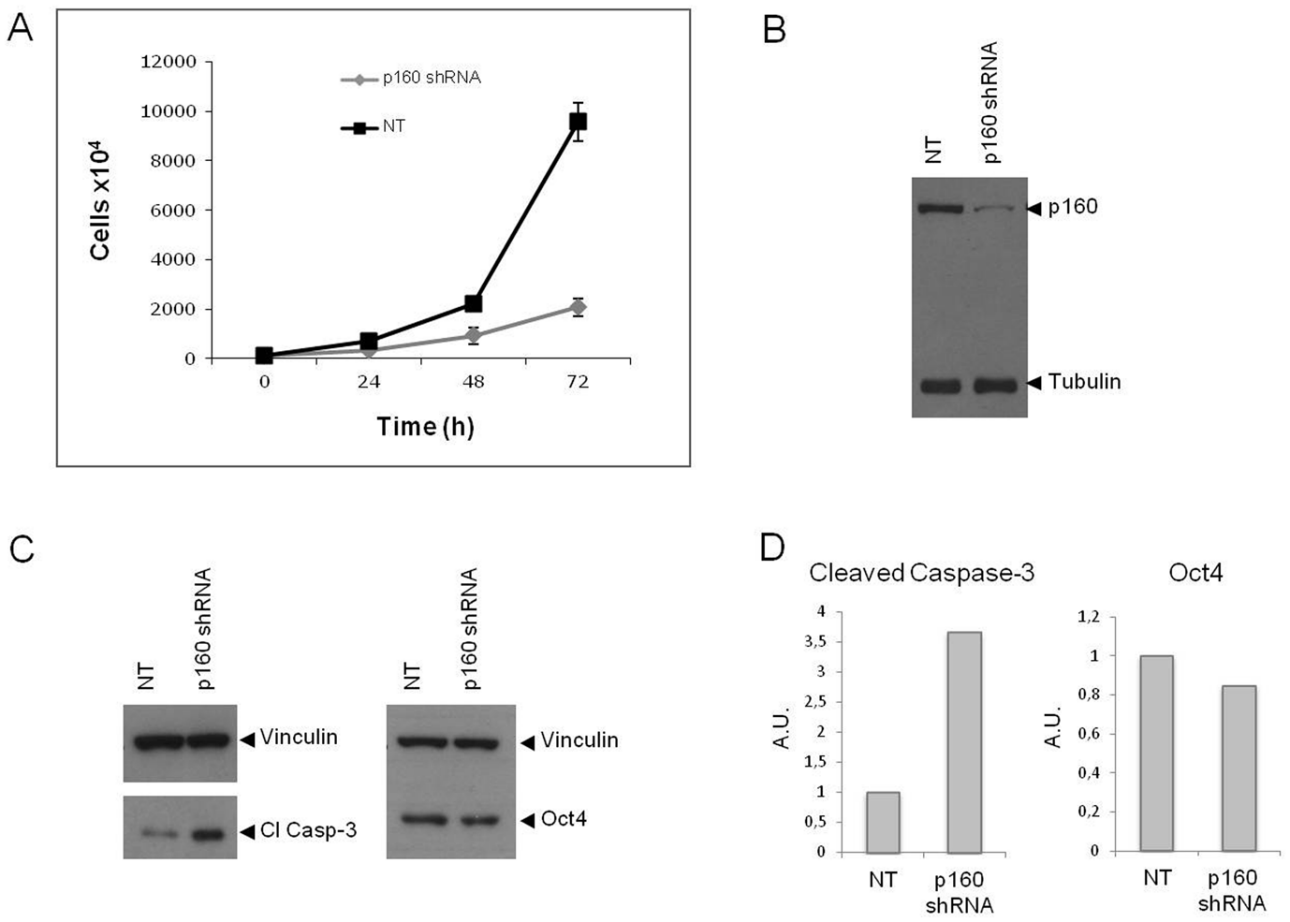
Specific down-regulation of *Mybbp1a* in wild type ES cells blocks proliferation and induces activation of caspase-3. (**A**) ES cells were infected with the *Mybbp1a*-specific shRNA3 or with an empty non-target (NT) lentivirus vector (see Materials and Methods), and the proliferation rate was measured starting two days after infection (0 h) every 24 h, using a cell counter. (**B**). At the end of the experiment (72 h) the cells were lysed and the extract immunoblotted with Mybbp1a-specific antibodies (anti-p160C) using Tubulin as loading control. (**C, D**). The extracts were also immunoblotted against Oct4 (for specificity of the down-regulation) and cleaved-Caspase 3 for apoptosis using Vinculin as loading control.

To investigate the molecular determinants of this phenotype, we down-regulated *Mybbp1a* in wild type C57BL6 ES cells using shRNA technology (shRNA3, see Methods; see also below for the effect of other MYBBP1A shRNAs). Within 72 h of transduction, *MYBBP1A* shRNA3 retrovirus reduced Mybbp1a protein expression by 82% with respect to non-targeting shRNA (**Figure 1B**). After 72 hrs the number of ES cells was about five fold less than in the controls while the doubling time doubled (**Figure 1A**). Concomitantly, the level of active caspase-3 increased by 3.65 fold (**Figure 1C–D**), consistent with the decrease in cell number.

This drastic and sudden effect may be the cause of the early embryonic lethal phenotype. The survival of a (small) fraction of *Mybbp1a* -down-regulated cells (**Figure 1A**) is probably due to the incomplete down-regulation.

**Figure 2:**
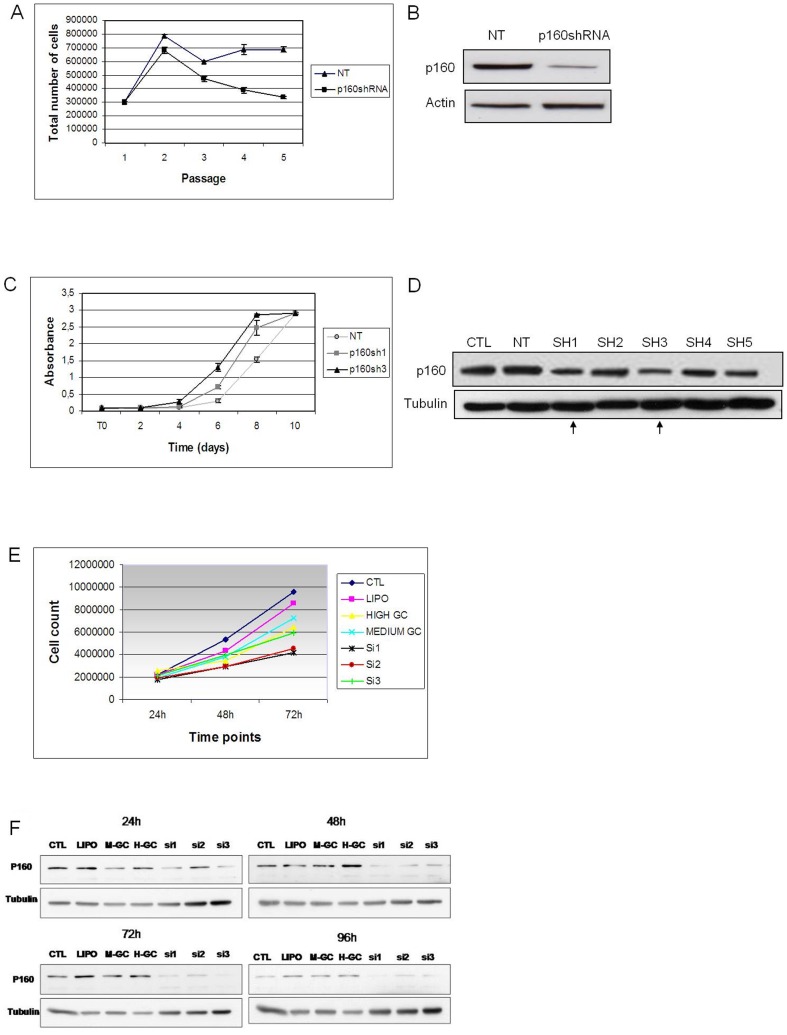
Down-regulation of *Mybbp1a* in primary wild type MEFs and in NIH3T3 cells has opposite effects on cell proliferation. (**A**) Down regulation of *Mybbp1a* in MEFs with the specific lentiviral vector shRNA3 induces early senescence, as opposed to a non-target empty (NT) vector. (**B**) Immunoblot of the cells of panel A showing down regulation of MYBBP1A (p160), using tubulin as control. (**C**) Growth rate determination (crystal violet assay, see Methods) in Mybbp1a down-regulated NIH3T3 cells with lentiviral vectors expressing specific Sh1RNA or Sh3RNA, as opposed with control empty Non Target vector (NT). Crystal violet assays were performed over 10 days counting the cells in triplicate every 2 days. The p-value of the difference between Sh3 and NT-treated cells was ≤0.001 (t test). (**D**) Immunoblot of the cells of panel C. (**E**) Growth rate determination of *MYBBP1A* down-regulated HeLa cells by specific siRNAs (see Methods). Ctl: untreated cells. Lipo: transfection control with lipofectamine only. High GC and Medium GC: two control oligonucleotides (indicated by the siRNAs manufacturer) of high and, respectively, medium GC content. siRNA1, siRNA2 and siRNA3: specific MYBBP1A siRNAs (see Methods for sequences). (**F**) Immunoblots of the cells of panel B upon culturing for 24, 48, 72 and 96 h after transfection.

To test the generality of this effect, we have down-regulated *Mybbp1a* in other mouse cells (MEFs and NIH3T3) and in human HeLa cells. Primary MEFs (only two passages in culture) down-regulated for *Mybbp1a* showed a rapid entry into senescence compared with cells treated with non-targeting vector (NT) in a long term proliferation protocol (**Figure 2A,B**). In human HeLa cancer cells, *MYBBP1A* was efficiently down-regulated by three tested siRNAs, in particular siRNA-1 and -3 (see **Figure 2F** and Methods); these achieved about 80% down-regulation within 48 h of transfection. **Figure 2E** shows that cells transfected with any of the three siRNAs grew appreciably slower than controls (commercial oligonucleotides of High or Medium GC content, see Methods).

Surprisingly, a different result was obtained using NIH3T3 cells, which are immortalized cells that can be transformed by a single oncogene. In this case, down-regulation of *Mybbp1a* caused an increased growth rate as measured by the crystal violet assay; we noted however, that knock-down was less efficient in these cells (Figure **2C, D**).

Overall, these results show that both in most of the cell types tested, MYBBP1A is required for normal rates of cell proliferation. The different effect observed in NIH3T3 cells upon *Mybbp1a* knock-down (growth acceleration rather than inhibition) was surprising and will be discussed further below.

### *MYBBP1A* knock-down in HeLa cells induces apoptosis, blocks surviving cells in the G2/M phase and causes mitotic aberrations

**Figure 3:**
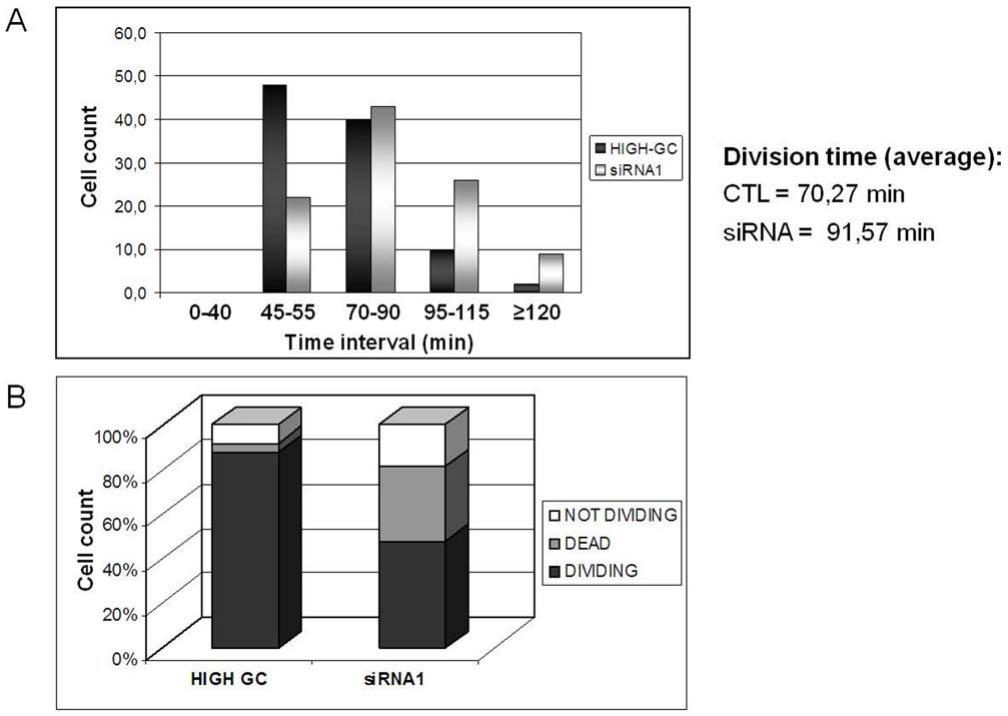
*MYBBP1A*-depletion slows the growth of HeLa cells. (**A**) Measure of the length of mitosis with Time-Lapse video imaging of HeLa cells transfected with High-GC or siRNA1 for 48 h after synchronization with a double thymidine block. The numbers below the histograms identify groups of cells with different mitosis length. Cells analyzed = 40 per group. (**B**) Percentage of dividing, not dividing or dead cells during the period analyzed by Time-Lapse microscopy; individual cells analyzed = 60.

We next investigated the basis for cell growth inhibition induced by *MYBBP1A* knock-down in HeLa cells. First, time-lapse experiments showed major mitotic abnormalities in *MYBBP1A*-down regulated HeLa cells. In most control cells the time required for mitosis was about 70 minutes but increased to an average of 92 after *MYBBP1A*-down regulation (**Figure 3A**). Examples are shown in **Suppl. Fig. S5**. In addition, 20% of the siRNA transfected cells did not divide at all (vs. 10% in Control) while about 40% died (4% in Control) (**Fig. 3B**).

**Figure 4:**
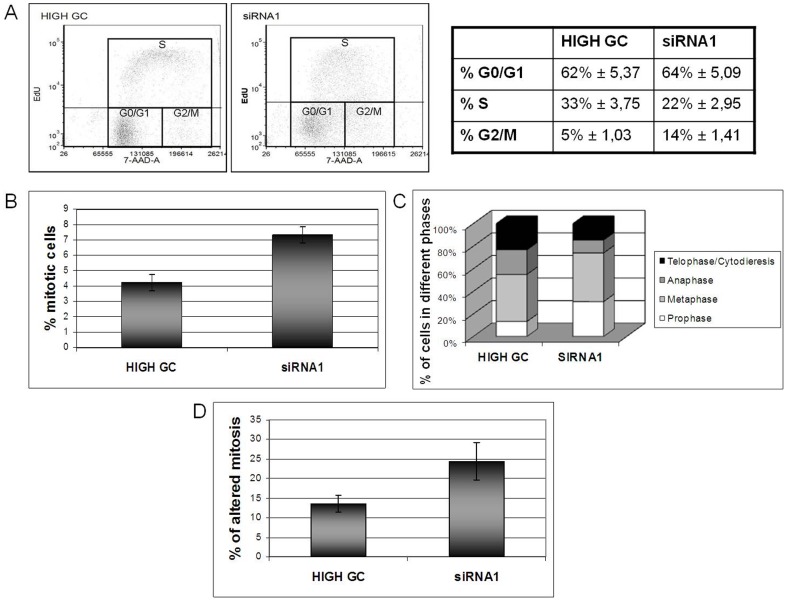
*MYBBP1A*-depleted HeLa cells are blocked in S/G2. (**A**) Flow cytometry and cell cycle analysis of HeLa cells after EdU incorporation using anti-EdU antibodies and 7-AAD. Flow cytometry histograms show cell cycle distribution and quantitation of HeLa cells transfected with High-GC or siRNA1 oligonucleotides, at 72 h post-transfection. (**B**) Quantitation of mitotic figures in control (High GC) and siRNA1 transfected HeLa cells performed with confocal microscope. (**C**) Quantitation of cells in the various phases of cell cycle in control (High GC) and siRNA1 transfected HeLa cells by confocal microscope. (**D**) Measurement of the percentage of anomalous mitotic figures in control (High GC) and siRNA1 treated HeLa cells by confocal microscope.

Consistent with this, we found that *MYBBP1A*-down regulated HeLa cells undergo a specific block of the cell cycle at G2/M. Flow cytometry for EdU and 7AAD (**Figure 4A**), in *MYBBP1A*-down-regulated HeLa cells, showed a 3-fold increase in the number of cells in G2/M (14 v. 5%) and a decrease in the number of cells in S phase, suggesting that the growth of the cells is delayed due to a block prior to cytokinesis. Interestingly (see also below) there may be a minor effect at the G1/S checkpoint (see Discussion). Upon direct microscopic observation, MYBBP1A-depleted cells contained in fact a much higher number of mitotic figures than controls (7% v. 4%, **Figure 4B**) but these were largely aberrant (**Figure 4D**); in particular, we observed altered metaphase plates and cytodieresis and multi-polar spindles (**Suppl. Fig. S6B**). No such aberrations were observed in the control cells (**Suppl. Fig. S6A**). These data suggest that MYBBP1A-down regulation causes a block/delay of cells in S/G2/M and that those cells that reached mitosis acquired major abnormalities. Finally, the number of cells in prophase increased at the expense of those in anaphase (**Figure 4C**).

**Figure 5:**
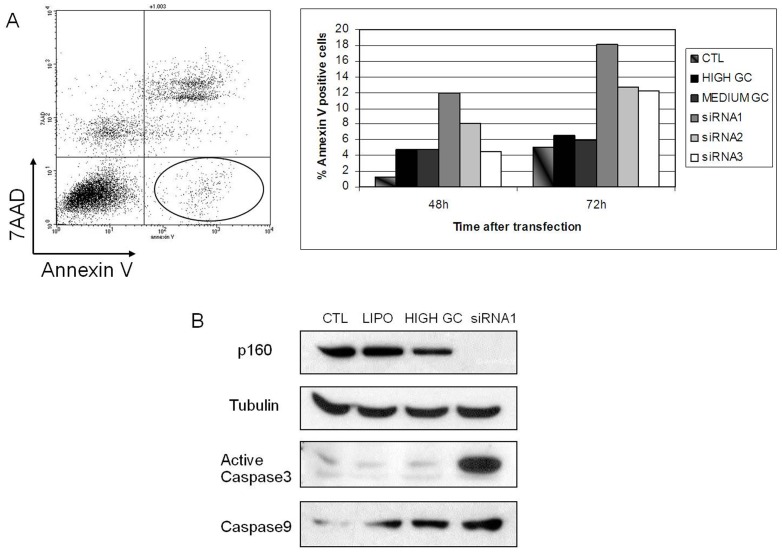
*MYBBP1A* down-regulation induces apoptosis. (**A**) HeLa cells were transiently transfected with siRNA1, 2 or 3, or with control High-GC or Medium-GC oligonucleotides, or with Lipofectamine only, for 48 h. The figure shows the determination of early apoptotic cells by flow cytometry (i.e. Annexin V positive and 7AAD-negative) (left panel, circled gate) 48 h post transfection. The histogram on the right shows the quantification in the various samples at the different times after transfection. In untreated cells only 1% of the cells were in apoptosis. (**B**) Western-blot analysis of HeLa cells transiently transfected for 48 h with CTL (untreated cells), LIPO (treated only with lipofectamine), HIGH GC (transfected with High-GC control), siRNA1 (transfected with MYBBP1A-specific siRNA1). The immunoblot was performed against MYBBP1A, active Caspase 3 and Caspase 9; tubulin is shown as loading control.

*MYBBP1A*-down regulation in HeLa cells also induces apoptosis. Indeed at 48 and 72 hrs, flow cytometry with Annexin-V vs. 7AAD showed about 10% and 15% early apoptotic cells (Annexin V-positive, 7AAD-negative), respectively, upon siRNA-transfection, compared to control transfection (about 6%) (**Figure 5A**). This was confirmed by immunoblotting with anti-active caspase 3 and anti caspase-9 antibodies (**Figure 5B**). In fact, the percent of Annexin-positive/7AAD-positive cells also increased upon *MYBBP1A* down-regulation (50+/−10% at 48 h and 80+/−30% at 72 h) (data not shown). This suggests an even greater effect on cell death.

### At mitosis, MYBBP1A assumes a para-chromosomal localization

**Figure 6:**
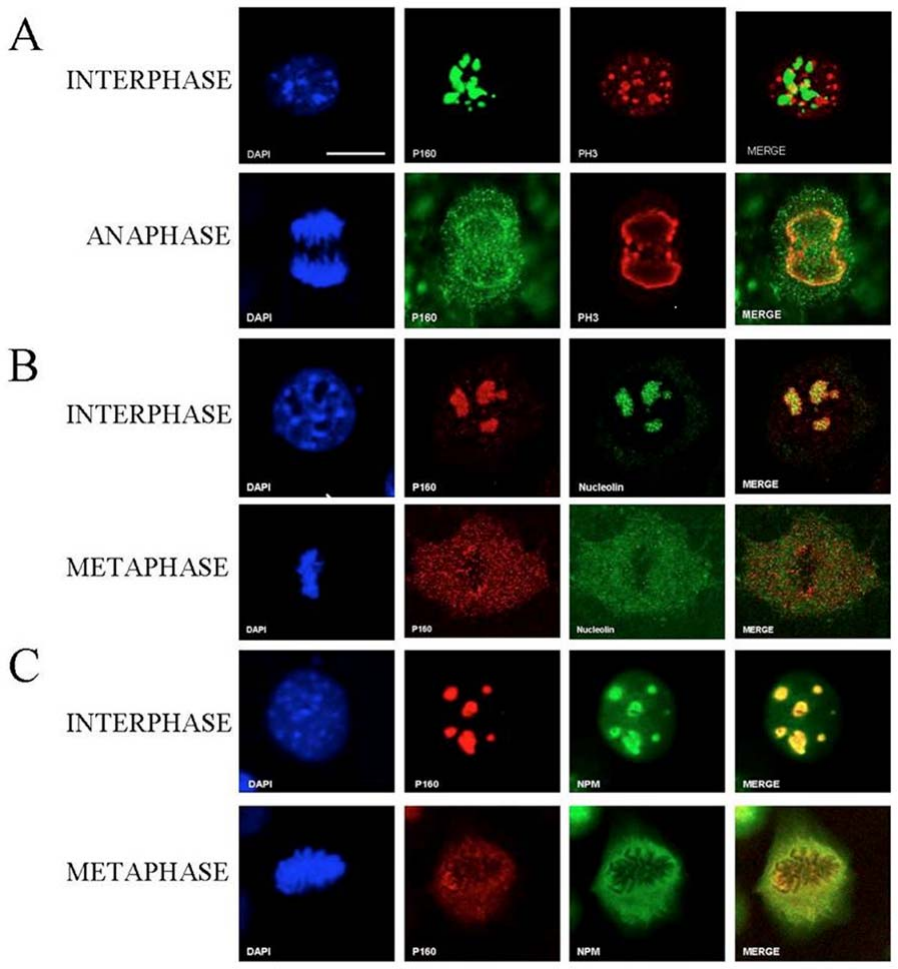
Mybbp1a localization during mitosis. Representative confocal images of NIH 3T3 cells stably expressing Mybbp1a-Flag in the cell-cycle phases indicated on the left. (**A**) The staining was performed with anti-Flag (p160, green), anti-Phospho-Histone-H3 (PH3, red) and DAPI (blue) for DNA. (**B**) Staining with anti-Flag (p160, red), anti-Nucleolin (green) and DAPI (blue) for DNA. (**C**) Staining with anti-Flag (p160, red), anti-Nucleophosmin (NPM, green) and DAPI (blue) for DNA. Bar = 5 µm. The magnification is the same in all panels.

The growth inhibition, extension of mitosis and G2/M block upon down-regulation of *MYBBP1A* all point to a possible involvement of MYBBP1A in mitosis. Since nucleoli are disrupted at mitosis we examined the localization of the nucleolar protein MYBBP1A in various phases of mitosis. **Fig. 6B, C** show that in NIH3T3 cells expressing Mybbp1a-flag, Mybbp1a co-localized with Nucleolin and Nucleophosmin (NPM) in nucleoli (see below). However, in metaphase and anaphase Mybbp1a was found in the nucleoplasm, particularly concentrated around the chromosomal plate. Nucleolin and NPM also exit the nucleolus at mitosis and co-localized with MYBBP1A at interphase; however, at metaphase, NPM (but not nucleolin) and Mybbp1a largely colocalized in the para-chromosomal regions. **Suppl. Figure S2B** shows that the distribution of Mybbp1a is not due to overexpression, since the same pattern is observed when endogenous Mybbp1a is examined in non-overexpressing cells with an antibody recognizing only the full-length protein.

At metaphase and anaphase, immunofluorescent staining (IF) showed that Mybbp1a co-localized with phospho-histone H3 (**Figure. 6A**), a chromatin marker. No co-localization of Mybbp1a was seen with inner kinetochore components (like constitutive centromere proteins CENP-A, -B, and –C) as shown by IF with anti-CREST antibodies, nor with telomeres, as shown by Immuno-FISH with a PNA probe (**Suppl. Fig. S3**).

From these data we conclude that Mybbp1a translocates from nucleoli to the nucleoplasm during mitosis, and in metaphase-anaphase is found spread throughout para-chromosomal regions, in apparent colocalization with a chromatin marker.

The same results were obtained with other cell lines, e.g. HeLa cells (See **Suppl. Figure S4**) where MYBBP1A again assumes a parachromosomal localization. Hence the differential effect of MYBBP1A down-regulation in HeLa v. NIH3T3 cells is not due to a different mitotic localization.

### MYBBP1A down-regulation affects the expression of cell cycle and growth controlling genes

Gene expression profiling of control and siRNA down-regulated HeLa cells (two biological duplicates) was carried out on an Affymetrix Gene ST 1.0 Human platform. Of the 28829 genes analyzed, 853 were differentially expressed (increased or decreased more than two fold: (*p*<10^−4^); 71.4% of the genes were down-regulated and 28.6% up-regulated upon *MYBBP1A* silencing (**Table 2**). Functional annotation clustering of the differentially expressed genes with the DAVID software (http://david.abcc.ncifcrf.gov/) shows that 49.8% of these genes fall into categories involved in cell-cycle, mitosis, DNA-damage and stress response, apoptosis, chromosome segregation and chromatin (**Table 3**).

**Table 2:** Genes differentially expressed in *MYBBP1A*-down-regulated HeLa cells*.

	N (%)
Total Number of detected genes on chip	28829
Total Number of differentially expressed genes§	853 (2.96%)
Up-regulated genes	244 (28.6%)
Down-regulated genes	609 (71.4%)

* Data obtained with a human Affymetrix ST 1.0 chip. Analysis of gene expression profiling on HeLa cells transfected with siRNA3, harvested 48 h after transfection.

§ Genes whose expression level varied by more than 50% (p<0.0001).

**Table 3:** Gene Onthology Analysis of the differentially expressed genes following *MYBBP1A*-down-regulation in HeLa cells.

GO Term	N. genes	%	*p-value*
**Cell cycle**	72	9.3	1.4E−10
**Mitosis**	27	3.5	1.3E−6
**Cell cycle arrest**	15	1.9	8.0E−5
**Chromosome segregation**	17	2.2	1.5E−7
**Sister chromatid exchange**	12	1.6	1.7E−7
**Chromosome condensation**	16	2.1	2.0E−4
**Spindle**	17	2.2	2.7E−4
**Response to DNA damage**	37	4.8	1.8E−6
**Apoptosis**	46	6.0	7.8E−5
**Microtubule organizing center**	25	3.2	8.1E−5
**Centrosome**	23	3.0	9.6E−5
**Chromatin**	21	2.7	1.5E−4
**Cellular response to stress**	52	6.7	1.1E−7

N = total number of differentially expressed genes within the GO cathegory.

%: percent of the total number of differentially expressed genes.

*p-value*: calculated on the basis of the enrichment of the category (ie number of genes affected/number of genes in the category).

Since our experiments suggested a growth-arrest at G2/M and genes of this pathway were the most enriched cell cycle genes in our data set (**Table 4**), we specifically validated this pathway by real-time PCR measurement of a subset of these genes (**Table 5**). In addition to the decrease of MYBBP1A itself, genes encoding major cell cycle or DNA damage-related proteins, were affected: CDKN1A, GADD45B and SFN were induced while TOP2A, TOP2B, BRCA1, CDK7 and WEE1 were down-regulated. This is in keeping with the above results and confirms that MYBBP1A affects cell-cycle regulation. Indeed, pathway analysis (Ingenuity Pathway Analysis, Ingenuity Systems) shows that in the G2/M pathway 3 out of 5 of the CDC2/CYCLINB2 activating genes are down-regulated (BRCA1, CDK7, WEE1) while 2 out of 3 of the inactivating ones (CDKN1A and GADD45B) are overexpressed (not shown). Importantly, among the validated genes, TOP2A, TOP2B, GADD45A and CDKN1A are also connected to mitotic dysfunction, in agreement with the data of **Figure 4** and **Suppl.** **Figure 6**.

**Table 4:** Validation by Q-PCR of the gene expression profile in *MYBBP1A*-depleted HeLa cells. #.

Gene	Ratio*
CKS1	0.977+/−0,085632938***
GADD45B	4.88+/−0,886155931***
CDKN1A (P21)	10.45+/−0,449326533***
SFN	1.29+/−0,088121129
NFKB2	2.9+/−0,0675**
cJUN	3.05+/−0,246***
MYBBP1A	0.09+/−0,00665***
TOP2A	0.27+/−0,014977761***
TOP2B	0.21+/−0,030892286***
WEE1	0.81+/−0,144244006**
BRCA1	0.35+/−0,029816103***
CDK7	0.76+/−0,097858742**

# Real-Time PCR was performed on HeLa control (High-GC) or *MYBBP1A*-depleted HeLa cells (siRNA1) with the same RNA samples used for microarray experiment. Two biological replicates were used for each condition and the experiment was performed in triplicate; an arbitrary value of 1 was given to the control (CTL).

* Ratio between the expression in down-regulated vs. control (see Methods) cells.

** p≤0.01.

*** p≤0.001 (t test).

**Table 5:** Tumorigenesis by RasVal12 in NIH3T3 cells infected with an empty vector (C) or with a retrovirus carrying an shRNA specific for *MYBBP1A* (Sh3).

	N#	Tumor Volume○+/−SEM* at day = 18	*p-value* $
C	6	0	
Sh3	6	0	
C+RasV12	6	1.75+/−0.22	
Sh3+RasV12	6	2.43+/−0.23	0.048

# Total number of mice in the group.

○ volume expressed in cm^3^.

* SEM = Standard Error of the Mean.

$ Student's t-test.

### Reduction of MYBBP1A enhances the oncogenic activity of Ras^Val12^

Since the down-regulation of *MYBBP1A* affected cell growth and chromosomal stability, we considered whether it might also affect the activity of oncogenes. We therefore tested the effect of *Mybbp1a* down-regulation in the mouse NIH3T3 cell line which is particularly suited to tumorigenesis studies, and in which Mybbp1a down-regulation accelerated rather than inhibited cell growth (**Figure 2C**). As shown above, vectors containing shRNA3 or shRNA1 down-regulated Mybbp1a on average by about 60% (see **Fig. 2D**).

**Figure 7:**
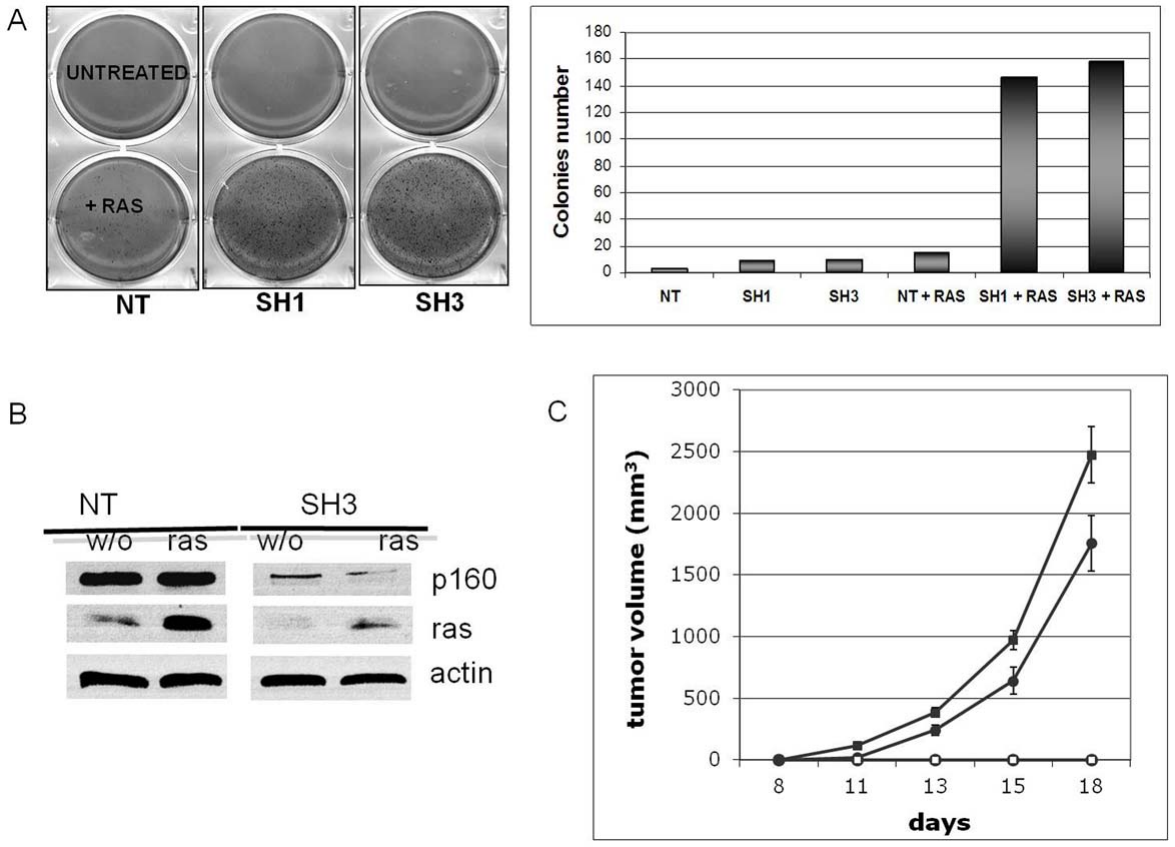
Down-regulation of *Mybbp1a* in NIH3T3 cells favors transformation by RasV12. (**A**) Soft agar colony assay with NIH 3T3 cells infected with non-target vector (NT), shRNA1 or shRNA3 lentiviruses with or without co-infection with pBABE-Ras^Val12^ retrovirus. 50,000 cells were plated in triplicate in soft agar and the images recorded 9 days after plating. The left panel shows examples of the actual plates, the right panel the quantification of the experiment. (**B**) Immunoblot analysis of the levels of Ras and Mybbp1a after infection with pBABE-RavV12 and SH3 or NT (empty, non target control) lentivirus, respectively. The apparent difference in the level of Ras in this blot is due to a difference in loading (see the actin band). (**C**) Enhanced tumor growth rate upon Mybbp1a depletion. Cells were injected in the flank of nude mice (n = 6/group) and the size of the tumor evaluated at different times thereafter (ordinates, days). NIH3T3 cells were infected with non-target empty lentivirus vector (NT) alone (○), non-targeting lentivirus vector (NT) plus pBABE-RasV12 (•), *Mybbp1a*-specific shRNA3 lentivirus vector alone (□) or *Mybbp1a*-specific shRNA3 lentivirus vector plus pBABE-RasV12 (▪). At each time point, the Student's t-test gave a p-value of 0.0188 (11 days), 0.0334 (13 days), 0.035 (15 days) and 0.048 (18 days). This panel shows a single experiment, representative of a total of three, with the same result.

When co-infected with a HaRas^Val12^ retrovirus, NIH3T3 cells formed a higher number of colonies in soft agar than controls (**Fig. 7A**), suggesting that decreased Mybbp1a favors Ras transformation. Therefore, we also tested whether MYBBP1A down-regulation affected the tumorigenic activity of control and Ras-transformed NIH-3T3 cells upon injection into nude mice. As shown in **Figure 7C**, *Mybbp1a* down-regulated NIH-3T3 cells did not induce tumor formation on their own. However, Ras-transformed, *Mybbp1a*-down-regulated NIH-3T3 cells showed significantly accelerated tumor formation in nude mice compared to control Ras-transformed cells. The results therefore indicate that Mybbp1a has a tumor-suppressive function.

## Discussion

The present data define an essential role of MYBBP1A in cell cycle and mitosis, as its absence/down-regulation causes chromosomal defects, G2/M arrest, mitotic anomalies and enhances the tumorigenicity of Ras. We have examined the function of MYBBP1A *in vivo*, in primary mouse cells (ES and MEFs), in immortalized NIH3T3 and in cancer HeLa cells. In all cases, the deletion or down-regulation of MYBBP1A has had a profound effect on the growth rate of the cells.

*In vivo*, MYBBP1A deletion leads to embryonic death at the blastocyst stage. Further analysis is required to determine what is occurring in the mutant embryos and whether cell division defects, as observed in the cell lines tested here, are responsible. Gene expression analyses of human *MYBBP1A* down-regulated HeLa cells shows altered expression of genes involved in chromosome segregation, kinetochore formation and, in general, mitosis. The involvement of MYBBP1A in mitosis is also directly suggested by its peri-chromosomal localization during metaphase (**Figure 6**). Indeed, MYBBP1A is phosphorylated by Aurora B at serine 1303 in nocodazole-arrested cells and is dephosphorylated upon Aurora B silencing or by treatment with Danusertib, a small molecule inhibitor of Aurora kinases [14]. It is therefore possible that MYBBP1A functions downstream of the Aurora B pathway, possibly in kinetochore formation and or chromosome segregation.

Down-regulation of MYBBP1A in normal primary cells like ES and MEFs has an effect superimposable to that on human cancer HeLa cells, i.e. a strong decrease in growth rate and increased apoptosis. In HeLa cells, surviving cells are blocked at the G2/M stage of the cell cycle. The gene expression analysis highlights the concordant mis-expression (increase of inhibitors, decrease of activators) of genes important in cell cycle regulation. In addition, this analysis revealed, as mentioned above, genes that are important in chromosomal segregation and kinetochore formation. It appears therefore, that MYBBP1A affects two important steps of cell division: the cell cycle and mitosis.

*Mybbp1a* down-regulation in NIH3T3 cells, however, has a different effect - stimulating rather than blocking growth, and enhancing Ha-RasV12 tumorigenesis. In these cells, the shRNA constructs were somewhat less efficient, in general causing about 60% decrease in Mybbp1a levels (**Fig. 2D**). It is not clear whether the different behavior of NIH3T3 cells is linked to this less complete down-regulation or to the presence of mutations accumulated during the long culture period in the laboratories. NIH3T3 cells are clearly different from primary cells: for example, they can be transformed by a single oncogene instead of two. This is due to the presence of mutations which have led to immortalization. Yet in this case, *Mybbp1a* down-regulation still affects cell proliferation, although in the opposite direction. It will be interesting to screen NIH3T3 cells for genes modifying Mybbp1a function and to see which are affected by MYBBP1A knock-down. The NIH3T3 transformation data also suggest that *Mybbp1a* might be able to act as a tumor suppressor gene in some contexts. Indeed, Mybbp1a possesses the important properties of controlling cell cycle and tumorigenicity of the Ras oncogene. A tumor-suppressor function is in line with all the results reported in this paper. Interestingly, *MYBBP1A* maps to human chromosome 17p13.3 in a region that is frequently translocated in human cancers (http://cgap.nci.nih.gov/Chromosomes/Mitelman).

**Figure 8:**
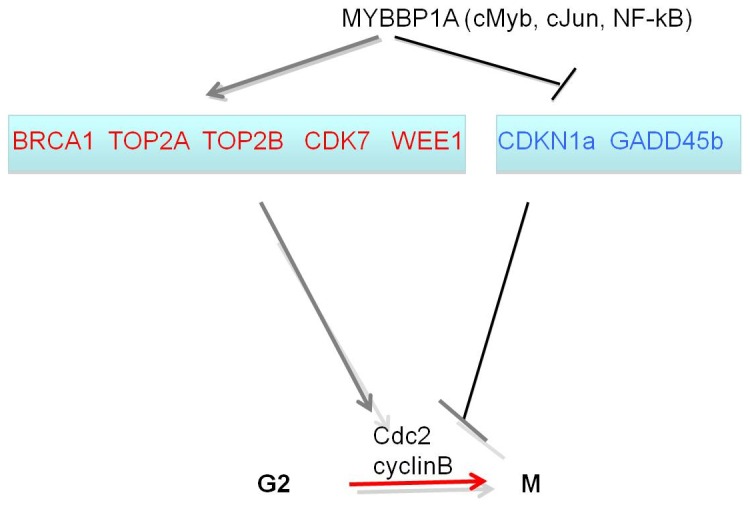
Connection between *MYBBP1A*, cell cycle genes and their function. The figure depicts the connection between *MYBBP1A*, the genes regulating the cell cycle that are altered by *MYBBP1A* down-regulation and their function. In particular, the expression of red genes is increased in the absence of MYBBP1A, while those in blue are decreased. Thus, MYBBP1A induces the inhibitors of Cdc2/cyclinB and down-regulates the activators. Note that although the figure focuses on the G2/M phase, some of the genes function also at the G1/S (in particular CDKN1A). MYBBP1A might act by regulating the synthesis and/or activity of one or more of its partner transcription factors (cMyb, cJun, NF-kB). In fact, the level of cJun mRNA is increased upon *MYBBP1A* down-regulation (Table 4).

In HeLa cells, a strong down-regulation of *MYBBP1A* affected the expression of several genes involved in cell cycle control, DNA damage and cell death (**Table 4**, **Figure 8**). In particular, genes inhibiting growth were up-regulated while genes important in DNA repair were down-regulated. These effects converge on the regulation of the Cdc2-cyclinB kinase complex (**Fig. 8**) and hence of the G2/M phase. In normal cells CDKN1A (p21) induces senescence [27] while GADD45 induces G2/M block [8]. On the other hand, BRCA1, the two topoisomerases TOP2a-TOP2b which are essential in DNA repair, and Wee1 which stimulates the G2/M phase were down-regulated. Since in HeLa cells p53 is inactivated by HPV E6, the upregulation of CDKN1A suggests that MYBBP1A prevents the transcriptional induction of CDKN1A by a p53-independent mechanism.

The down-regulation of *MYBBP1A* induces also a variety of mitotic anomalies which are not observed in control cells (**Figure 4** and **Suppl. Figure S6**). Analysis of the genes affected by the *MYBBP1A* down-regulation (**Table 3**) shows alterations in the expression of another very relevant set of genes directly involved in mitosis, chromosome condensation, segregation, sister chromatid exchange, formation of the spindle, microtubule organizing center and centrosome. In fact, also some of the validated genes (CDKN1A, TOP2A, TOP2B, GADD45A) have been directly linked to mitosis.

In summary, our data indicate that *MYBBP1A* has at least two important functions which are essential for embryo development and cell proliferation: cell cycle and chromosome segregation control. At the same time it is clear that other as yet undetermined proteins mediate the function of MYBBP1A. Transformation protein p53 might be one of them since MYBBP1A modulates p53 activity. However p53 is inactivated in HeLa cells, which allowed us to investigate MYBBP1A in the absence of a functional p53. Yet, the genes affected by MYBBP1A and p53 are partly overlapping and thus it is possible that the pathways affected by the two proteins intersect.

It is interesting to notice that loss of the MYBBP1A partner *Prep1* [4] has a similar phenotype. *Prep1*-null embryos die very early in embryogenesis because they accumulate DNA damage; on the other hand, heterozygous or hypomorphic mice develop tumors [18], [28], [29]. Whether these two proteins also interact to regulate the functions described above remains to be established.

## Supporting Information

Figure S1 **Targeted disruption of a** ***Mybbp1a*** **allele.** (A) Targeting strategy. The wild type *Mybbp1a* locus spanning the coding region (18.2 kb) is depicted (top). The *Mybbp1a* targeting vector (see Materials and Methods) was constructed using Mybbp1a 5′ genomic sequences (3.5 kb) and 3′ sequences (6.0 kb) flanking a pgkNEO expression cassette (1.8 kb) as shown. The targeted allele maintains the 3′ region, but removes 5′ region including the initiation codon (ATG) and exons 1–13, as depicted (bottom). Non-coding sequences are shown as thick black lines, exons are black boxes and restriction enzyme sites are indicated as H, HindIII, and X, XbaI. (B) Genotyping of ES cells and mice. XbaI digestion of genomic DNA produces a 7.4 kb fragment detected by the 5′ probe that corresponds to the wild type locus and a 5.2 kb fragment that corresponds to the targeted locus. The region between the dashed lines corresponds to the region of the wild type locus that was replaced with the pgkNEO cassette in the targeted allele. The location of the 5′ and 3′ probes is shown. Restriction enzyme sites are indicated as H, HindIII and X, XbaI. (C) Southern blot of XbaI-digested genomic DNA from wild type (WT) and heterozygous *Mybbp1a*+/− mice. The 5′probe detects the 7.4 kb upper band corresponding to the wild type *Mybbp1a* allele and the 5.2 kb band corresponding to the targeted allele. (D) PCR genotyping of mouse embryos from *Mybbp1a*+/− intercrosses. Controls are in lane 1 (−), which lacks template and contains the 3 primers KO3, KO4 and KO5, and lanes 2 and 3 (C+/−), which is genomic DNA from an adult heterozygous mouse. The remaining lanes are material from blastocyst outgrowths harvested from *Mybbp1a*+/− intercrosses. The upper band of 570 bp corresponds to the targeted allele and the 488 bp lower band corresponds to the wild type allele. Each allele was detected in a separate reaction. M, DNA size markers.
(TIF)

Figure S2 **Mybbp1a redistribution at mitosis.** A. Representative immunofluorescence confocal images of NIH 3T3 cells stably expressing *Mybbp1a*-Flag (p160-Flag) in the mitotic phases indicated on the left. Cells were stained with antibodies against Flag (Mybbp1a, red), α-Tubulin (green) and DAPI (blue) for DNA. Bar = 10 µm. B. Immunofluorescence staining of endogenous Mybbp1a in NIH3T3 cells compared with the staining of the cells overexpressing Mybbp1a-Flag.
(PDF)

Figure S3 **Mybbp1a does not localize at the kinetocore and on telomeres.** Representative confocal immunofluorescence of NIH 3T3 cells stably expressing Mybbp1a-Flag in the cell-cycle phases indicated on the left of the panels. The staining was performed against Flag (Mybbp1a, green), CREST (red, panel A), PNA (red, panel B) and with DAPI (blue) for DNA. Bar = 5 µm.
(TIF)

Figure S4 **Immunofluorescence analysis of MYBBP1A localization at interphase (top) and metaphase (bottom) in HeLa cells.** MYBBP1A localizes in the nucleoli at interphase but around the chromosomes at metaphase.
(TIF)

Figure S5 **Cell Cycle defects in** ***MYBBP1A*** **-depleted HeLa cells.** Four examples of time-lapse microscopic analysis, showing the widespread variation of the time required for mitosis after *MYBBP1A*-specific downregulation with si1 siRNA in HeLa cells. *MYBBP1A*-silenced cells have an extended mitotic period. Wide-field Time-Lapse video imaging of HeLa cells transfected with siRNA1 for 48 hours and synchronized with double thymidine block. Images were taken every 5 minutes starting 8 hours after the release from the block (mitosis start) with Oko-Vision Time Lapse microscope for a total of 18 hours. Four different examples are provided.
(TIF)

Figure S6 **Representative mitotic alterations caused by MYBBP1A depletion.** Confocal microscopy of control (A) and siRNA1 transfected (for 48 hrs) HeLa cells (B). In panel B: 1) multipolar spindle, 2) and 3) altered metaphasic plate, 4) triangular cytodieresis. Cells were stained with anti-Tubulin (green) antibodies to image the mitotic spindle and with DAPI (blue) for DNA. Bar = 12 µm.
(TIF)
