# Supplementary figures and images for: Myb-Binding Protein 1A (MYBBP1A) Is Essential for Early Embryonic Development, Controls Cell Cycle and Mitosis, and Acts as a Tumor Suppressor
Source: PLoS One. 2012 Oct 8;7(10):e39723. doi: 10.1371/journal.pone.0039723 (PMC3466261; doi:10.1371/journal.pone.0039723)

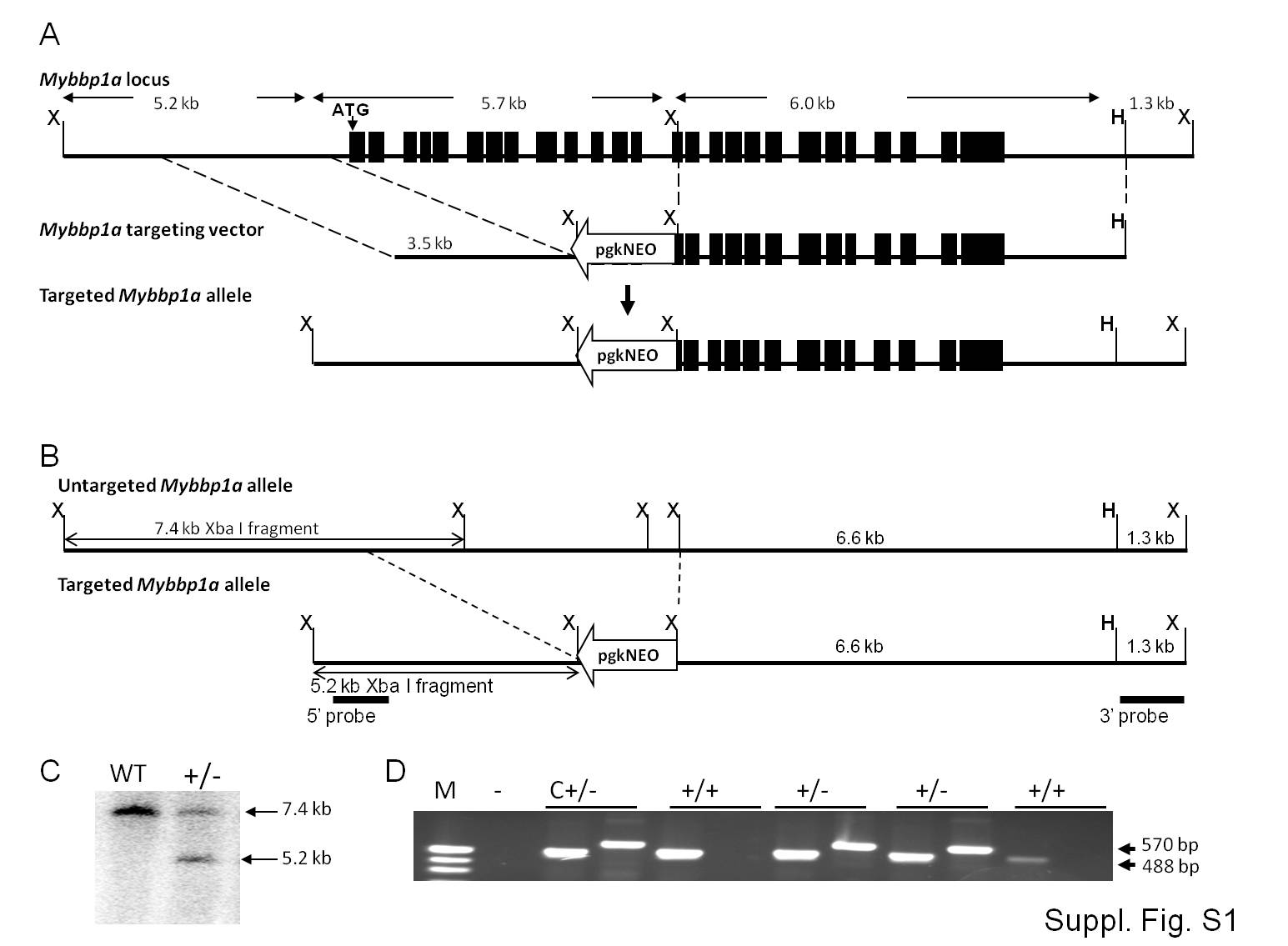

Supplement: Figure S1 — Targeted disruption of a Mybbp1a allele. (A) Targeting strategy. The wild type Mybbp1a locus spanning the coding region (18.2 kb) is depicted (top). The Mybbp1a targeting vector (see Materials and Methods) was constructed using Mybbp1a 5′ genomic sequences (3.5 kb) and 3′ sequences (6.0 kb) flanking a pgkNEO expression cassette (1.8 kb) as shown. The targeted allele maintains the 3′ region, but removes 5′ region including the initiation codon (ATG) and exons 1–13, as depicted (bottom). Non-coding sequences are shown as thick black lines, exons are black boxes and restriction enzyme sites are indicated as H, HindIII, and X, XbaI. (B) Genotyping of ES cells and mice. XbaI digestion of genomic DNA produces a 7.4 kb fragment detected by the 5′ probe that corresponds to the wild type locus and a 5.2 kb fragment that corresponds to the targeted locus. The region between the dashed lines corresponds to the region of the wild type locus that was replaced with the pgkNEO cassette in the targeted allele. The location of the 5′ and 3′ probes is shown. Restriction enzyme sites are indicated as H, HindIII and X, XbaI. (C) Southern blot of XbaI-digested genomic DNA from wild type (WT) and heterozygous Mybbp1a+/− mice. The 5′probe detects the 7.4 kb upper band corresponding to the wild type Mybbp1a allele and the 5.2 kb band corresponding to the targeted allele. (D) PCR genotyping of mouse embryos from Mybbp1a+/− intercrosses. Controls are in lane 1 (−), which lacks template and contains the 3 primers KO3, KO4 and KO5, and lanes 2 and 3 (C+/−), which is genomic DNA from an adult heterozygous mouse. The remaining lanes are material from blastocyst outgrowths harvested from Mybbp1a+/− intercrosses. The upper band of 570 bp corresponds to the targeted allele and the 488 bp lower band corresponds to the wild type allele. Each allele was detected in a separate reaction. M, DNA size markers. (TIF) [file pone.0039723.s001.tif]

A

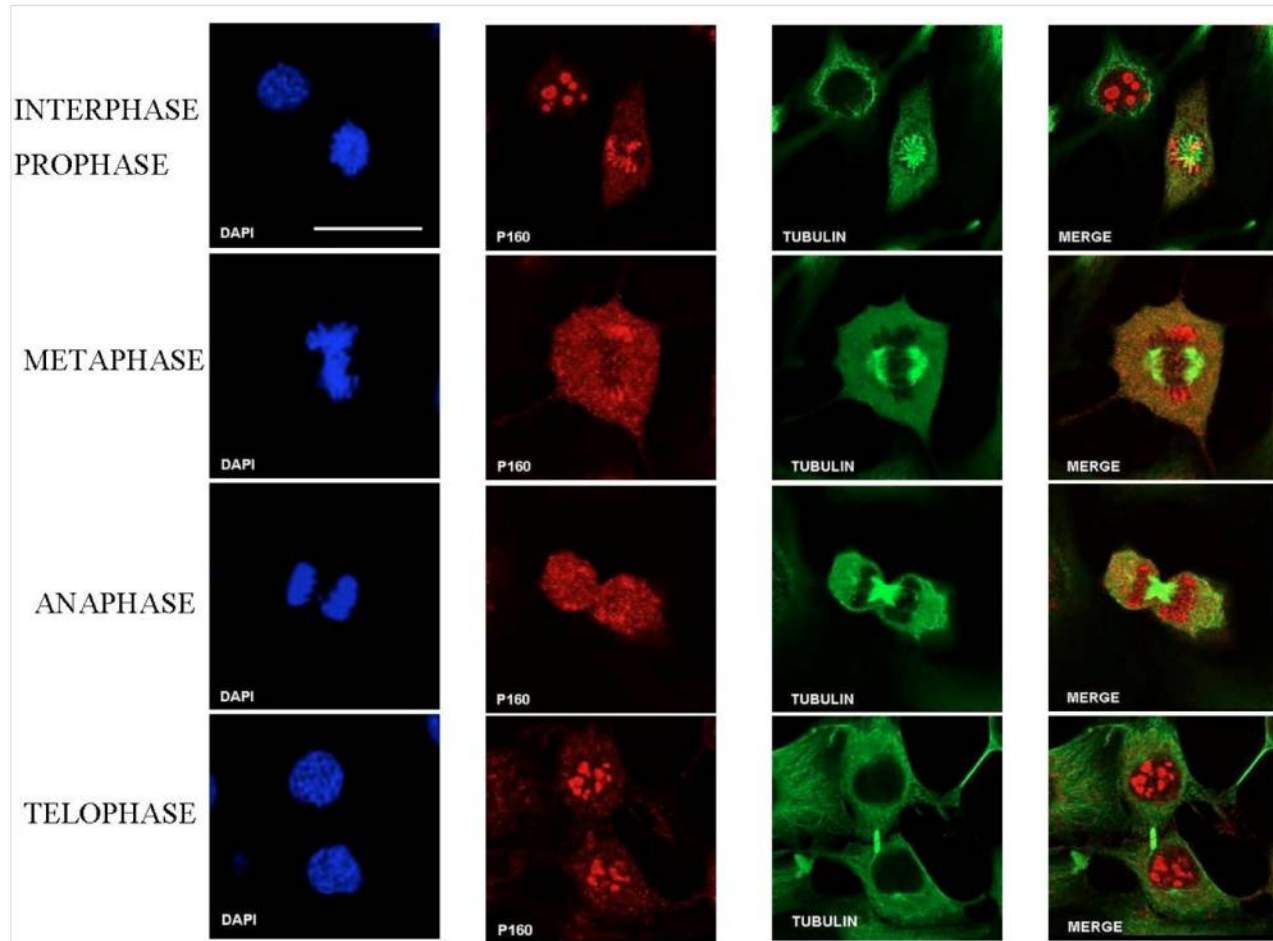

B

NIH 3T3 WT

NIH 3T3 p160Flag

Anti-p160

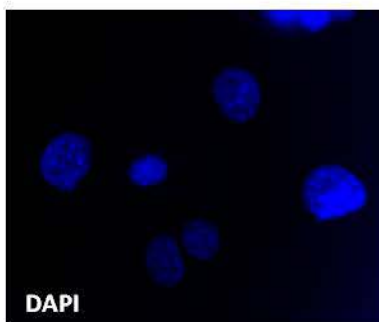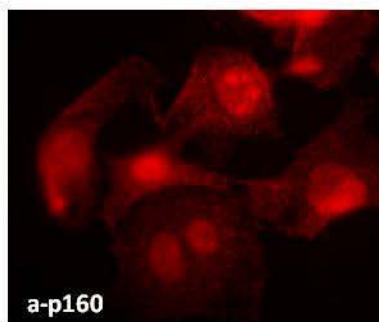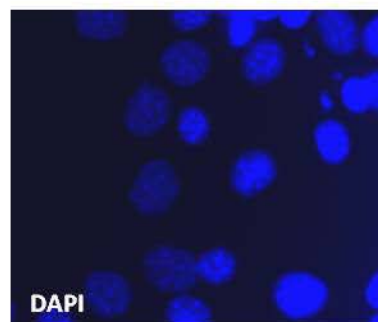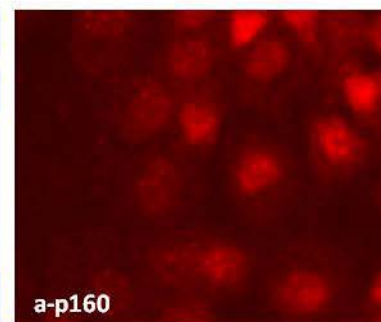

Anti-flag

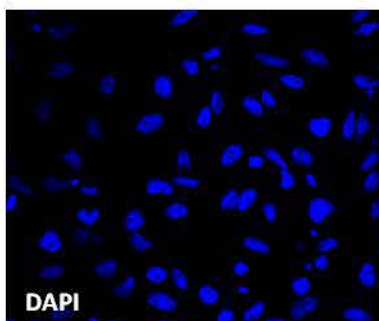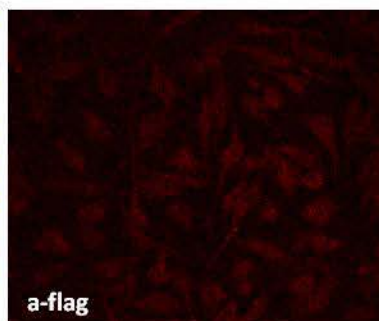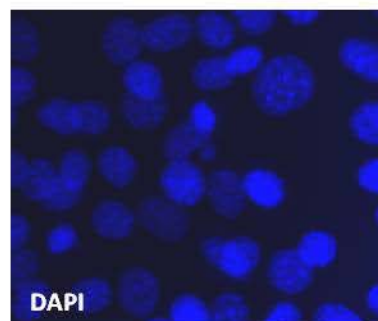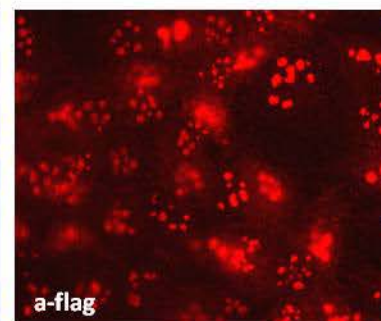

Supplement: Figure S2 — Mybbp1a redistribution at mitosis. A. Representative immunofluorescence confocal images of NIH 3T3 cells stably expressing Mybbp1a-Flag (p160-Flag) in the mitotic phases indicated on the left. Cells were stained with antibodies against Flag (Mybbp1a, red), α-Tubulin (green) and DAPI (blue) for DNA. Bar = 10 µm. B. Immunofluorescence staining of endogenous Mybbp1a in NIH3T3 cells compared with the staining of the cells overexpressing Mybbp1a-Flag. (PDF) [file pone.0039723.s002.pdf]

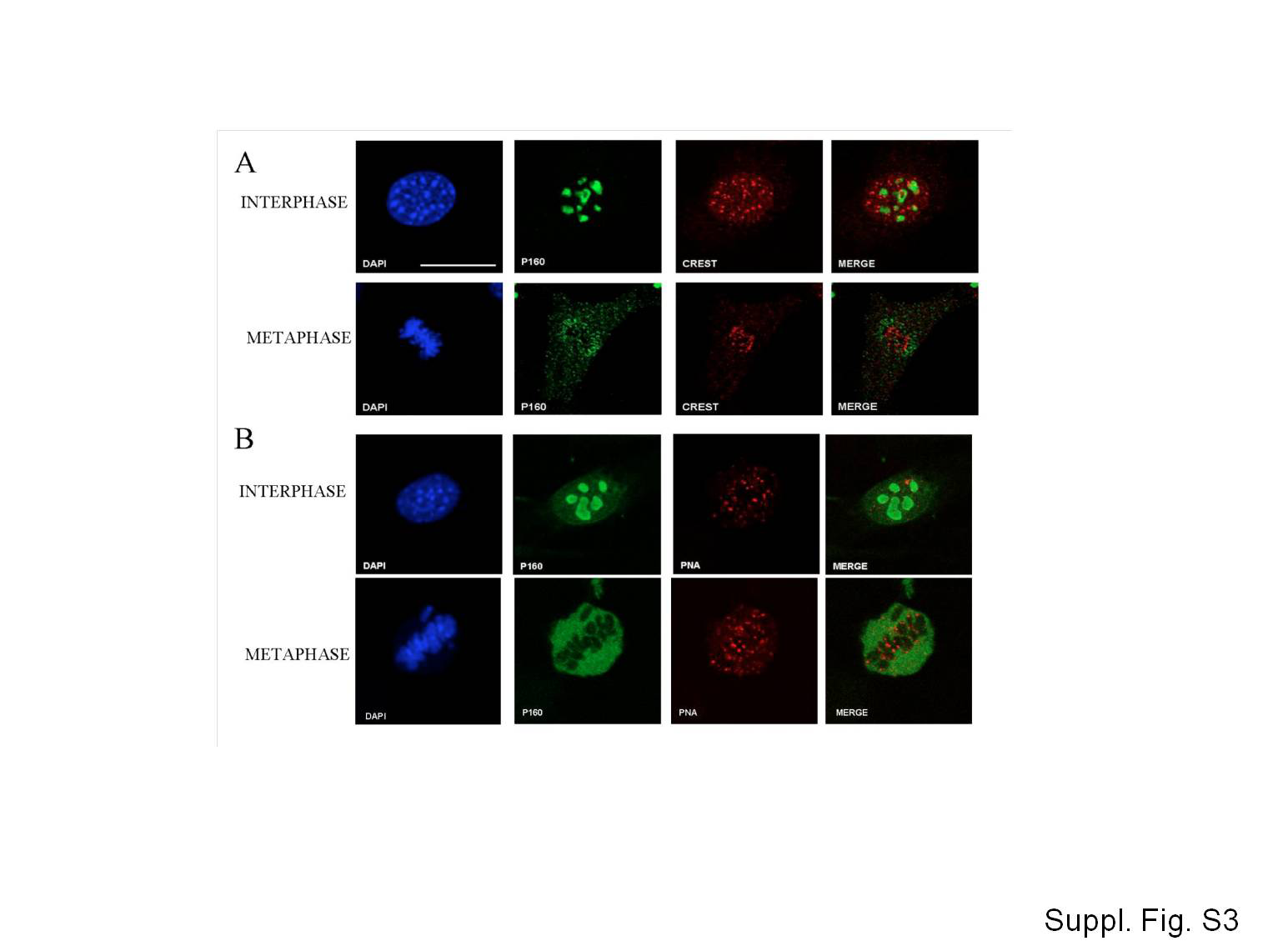

Supplement: Figure S3 — Mybbp1a does not localize at the kinetocore and on telomeres. Representative confocal immunofluorescence of NIH 3T3 cells stably expressing Mybbp1a-Flag in the cell-cycle phases indicated on the left of the panels. The staining was performed against Flag (Mybbp1a, green), CREST (red, panel A), PNA (red, panel B) and with DAPI (blue) for DNA. Bar = 5 µm. (TIF) [file pone.0039723.s003.tif]

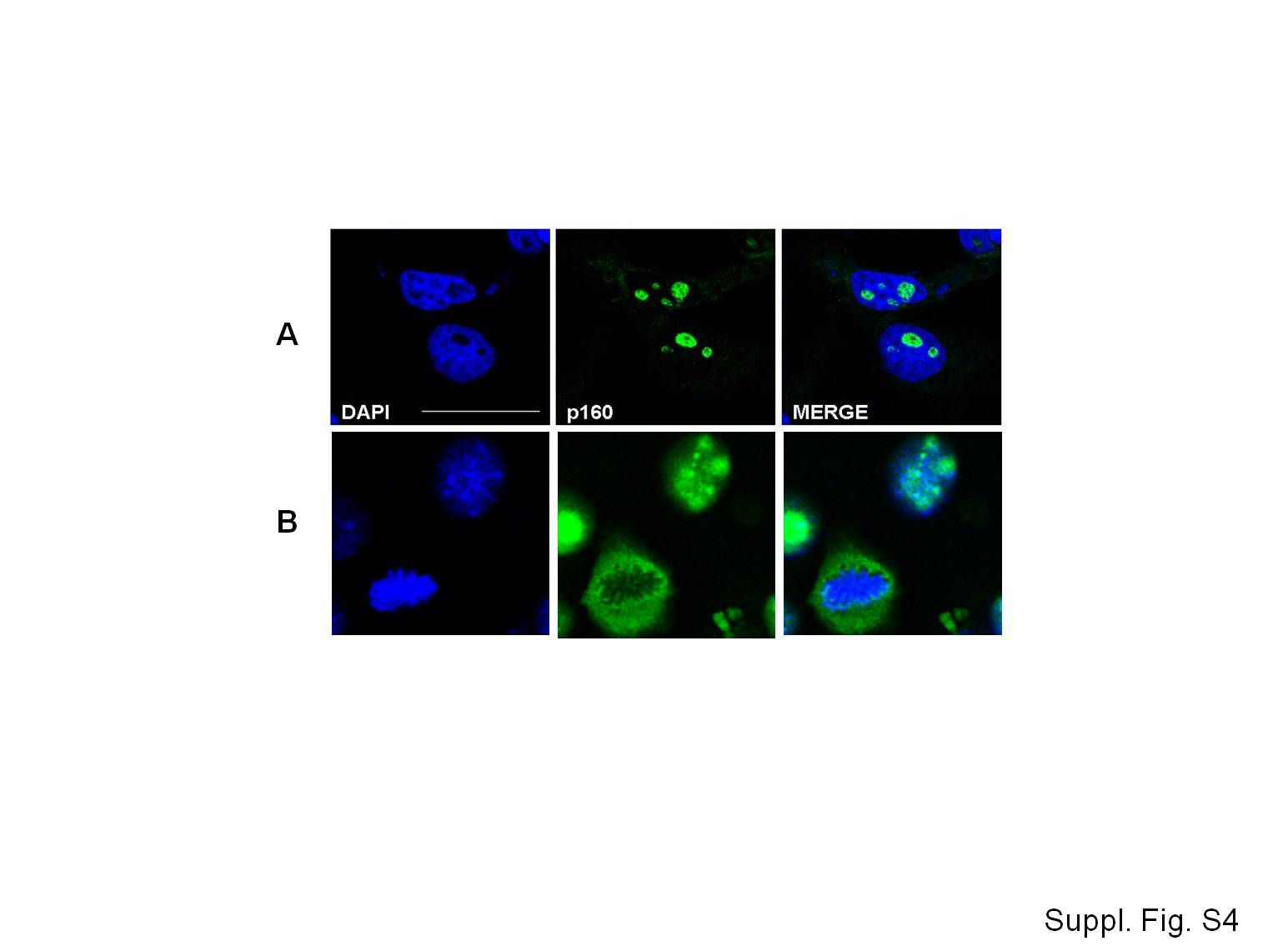

Supplement: Figure S4 — Immunofluorescence analysis of MYBBP1A localization at interphase (top) and metaphase (bottom) in HeLa cells. MYBBP1A localizes in the nucleoli at interphase but around the chromosomes at metaphase. (TIF) [file pone.0039723.s004.tif]

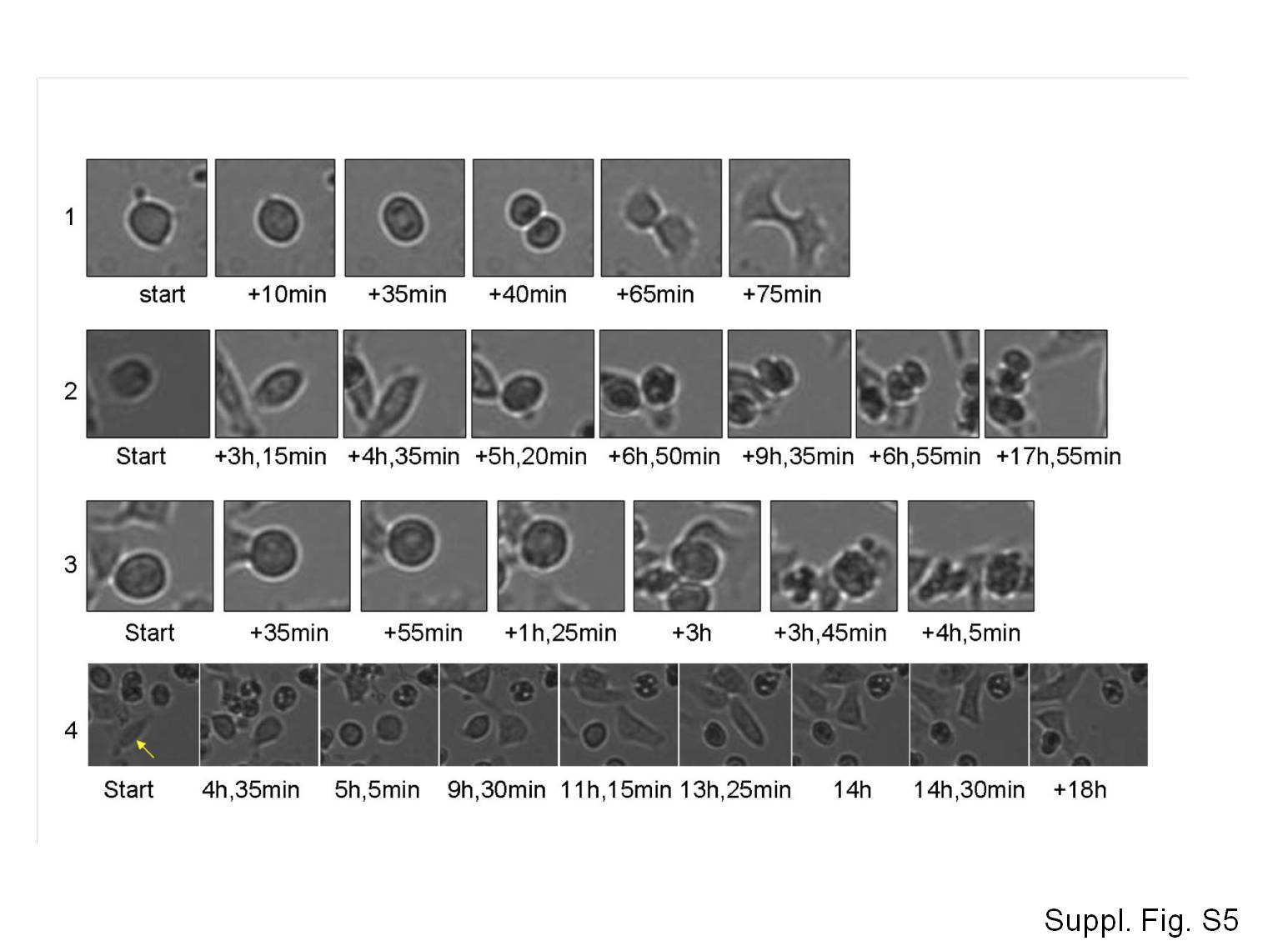

Supplement: Figure S5 — Cell Cycle defects in MYBBP1A -depleted HeLa cells. Four examples of time-lapse microscopic analysis, showing the widespread variation of the time required for mitosis after MYBBP1A-specific downregulation with si1 siRNA in HeLa cells. MYBBP1A-silenced cells have an extended mitotic period. Wide-field Time-Lapse video imaging of HeLa cells transfected with siRNA1 for 48 hours and synchronized with double thymidine block. Images were taken every 5 minutes starting 8 hours after the release from the block (mitosis start) with Oko-Vision Time Lapse microscope for a total of 18 hours. Four different examples are provided. (TIF) [file pone.0039723.s005.tif]

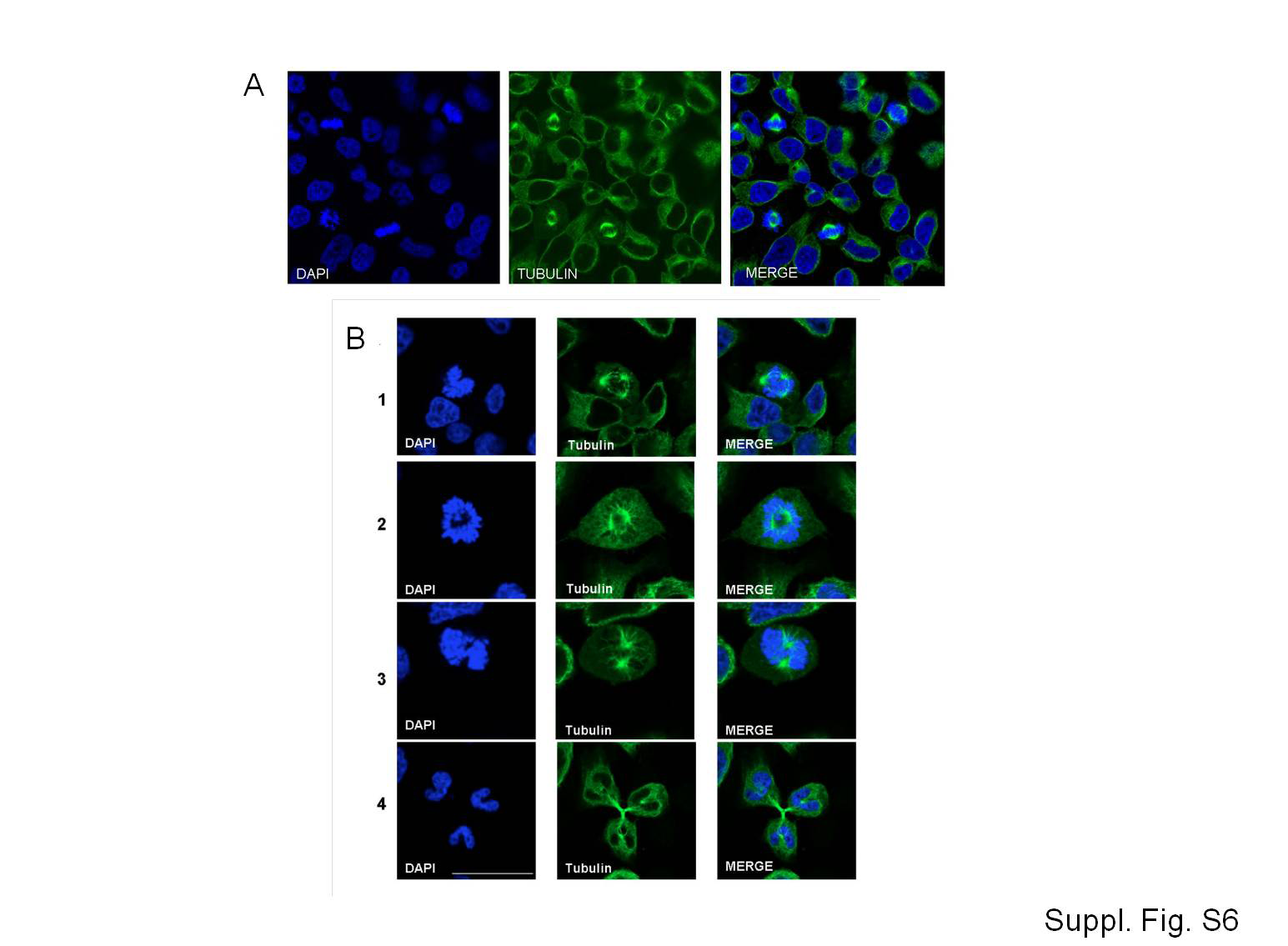

Supplement: Figure S6 — Representative mitotic alterations caused by MYBBP1A depletion. Confocal microscopy of control (A) and siRNA1 transfected (for 48 hrs) HeLa cells (B). In panel B: 1) multipolar spindle, 2) and 3) altered metaphasic plate, 4) triangular cytodieresis. Cells were stained with anti-Tubulin (green) antibodies to image the mitotic spindle and with DAPI (blue) for DNA. Bar = 12 µm. (TIF) [file pone.0039723.s006.tif]
